# Integrated Multi-Omics Analysis Reveals Molecular Features Associated with Energy Metabolism Adaptations in Brooding Taihe Black-Bone Silky Fowls

**DOI:** 10.3390/ani16152416

**Published:** 2026-08-05

**Authors:** Ramlat Ali Haji, Jing Hu, Jiming Ruan, Haiping Liang, Ziyue Wan, Salma Mbarouk Omar, Qing Wei, Xianhua Xie, Yanming Huang, Ji Cao, Jianzhen Huang

**Affiliations:** 1 College of Animal Science and Technology, Jiangxi Agricultural University, Nanchang 330045, China; ushahidihaji90@gmail.com (R.A.H.); liweiliu0824@gmail.com (J.H.); rjm903@163.com (J.R.); lianghaiping1967@126.com (H.L.); 13697911677@163.com (Z.W.); salmambarouk44@gmail.com (S.M.O.); octgrass@126.com (Q.W.); xiexianhua19880521@163.com (X.X.); 2 Department of Animal Science and Aquaculture, School of Agriculture (SOA), State University of Zanzibar (SUZA), Zanzibar P.O. Box 146, Tanzania; 3 Taihe Aoxin Wuji Development Co., Ltd., Taihe 343700, China; 15807968449@163.com

**Keywords:** brooding, energy metabolism, gut microbiota, liver, Taihe black-bone silky fowl

## Abstract

**Simple Summary:**

Broodiness is an inherent characteristic of female poultry, regulated by genetic, hormonal and environmental factors. This study was conducted using Taihe Black-Boned Silky Fowl (TBSF), a native Chinese chicken breed with a strong brooding tendency. The results indicate that brooding hens had significantly decreased feed intake and body weight, accompanied by altered lipid profiles in liver tissue and serum, as well as variations in hepatic gene expression, gut microbial pathways and serum metabolite levels. Furthermore, significant correlations were detected between gut microbial phyla, circulating metabolites and hepatic gene expression. Multiple lipid and amino acid metabolites were positively associated with specific microbes and genes responsible for lipid and amino acid synthesis. These findings deepen our understanding of key gut–liver axis metabolic targets regulating energy metabolism during broodiness, and provide strategies to improve reproductive performance and production efficiency in poultry.

**Abstract:**

Broodiness is a natural behavior whereby female birds stop laying eggs to sit on and hatch them. This behavior is regulated through genetic, hormonal, and environmental factors. The Taihe Black-Boned Silky Fowl (TBSF), a Chinese traditional domestic breed, exhibits a strong brooding tendency; however, the molecular mechanisms underlying this trait remain unclear. In this study, we performed an integrated multi-omics analysis to characterize differences between two groups of TBSF hens: 8 individuals undergoing 30 days of active brooding (BR30) and 8 individuals in the normal laying egg stage (NB), selected from a total group of 230 hens. We combined 16S rRNA sequencing, untargeted metabolomics, and hepatic transcriptome sequencing, with statistical analyses including QIIME 1.9.1, OPLS-DA (VIP > 1), Student’s *t*-test (*p* ≤ 0.05), and DESeq2 (|log_2_FC| ≥ 1, FDR < 0.05) for differentially expressed genes (DEGs), respectively, and Pearson’s correlation analysis for multi-omics integration. Phenotypically, brooder hens showed significantly reduced feed intake, body weight, and main digestive tissue indices, alongside altered liver and blood biochemical parameters. Hepatic transcriptome analysis identified 1582 DEGs between groups, enriched in pathways related to fatty acid oxidation and the amino acid degradation pathway. In addition, 16S rRNA sequencing revealed distinct gut microbial community structures: the NB group was enriched in *Bacilliota* and *Pseudomonadota*, while the BR30 group was enriched in *Spirochaetota* and *Synergistota*. Metabolomic profiling identified a total of 143 differential metabolites, which were enriched in lipid and amino acid metabolites, including alpha-linolenic acid and pyruvate metabolites. Multi-omics correlation analysis revealed tight associations between gut microbial taxa, circulating metabolites, and hepatic gene expression. Specifically, beneficial lipid metabolites, including phospholipids, lysophosphatidylcholines, and sphingomyelins, were positively correlated with *Synergistes* and the *Christensenellaceae R-7*, as well as with key hepatic lipid metabolism genes *FABP1*, *LPL*, and *FADS2*. In summary, this study reveals that the gut–liver axis plays a critical part in the modulation of energy metabolism during broodiness, and further highlights new insights into metabolic targets that could optimize reproductive behavior and enhance poultry production.

## 1. Introduction

Taihe Black-Boned Silky Fowl (TBSF), a renowned indigenous Chinese breed native to Jiangxi Province, is known for its strong natural brood instincts. Broodiness is a natural behavior shown by female birds influenced by a complex interplay of various factors, including genetic bias, hormonal regulation, and environmental cues. This behavior is characterized by the hen’s decreased voluntary feeding, cessation of egg laying, and persistent nesting to incubate a clutch of eggs [1,2]. The broodiness behavior triggers a substantial reduction in energy intake, prompting a critical metabolic shift from glucose utilization to lipid metabolism as the body’s energy reserves become depleted. For instance, Spratt et al.’s study in broiler breeder chickens demonstrated that such energy reduction significantly alters metabolism in the hepatic tissue, intestine loop, and reproductive tract [3]. Furthermore, research on Silky fowl indicates that Silky hens exhibit a stronger blood glucose recovery during starvation compared with other breeds [4,5]. However, no exact study was found on the precise phrase regarding brooding behavior [6]. Understanding the metabolic mechanisms underlying TBSF broodiness may therefore provide novel insights for improving energy management and production efficiency in poultry.

As a hub for communication with peripheral tissues, the liver plays a dynamic role in nutrient processing, transporting, storing, and metabolizing [7]. The liver and intestine are closely interconnected through the gut–liver axis, which facilitates bidirectional communication via microbial metabolites, bile acids, immune signals, and metabolic pathways to regulate systemic energy homeostasis. Nutrients absorbed in the gut are transported to the liver through the hepatic portal vein, where they are metabolized to supply energy for physiological needs [8,9]. Earlier research in humans revealed that high-fiber diets and beneficial bacteria optimize the gut–liver axis, leading to more efficient absorption and utilization of both nutrients and energy [10]. Additionally, alterations in gut microbial composition can significantly influence hepatic energy metabolism [11]. The gut–liver axis plays an indispensable role in energy regulation, and changes in gut microbiota are closely associated with host metabolic status. Further studies have shown that metabolites, such as bile acids and short-chain fatty acids (SCFAs), modulate key hepatic metabolism pathways, including gluconeogenesis, lipolysis, and ketogenesis, which are crucial in the gut–liver axis during both fed and fasting states [12]. In addition, the study by Zhu et al. revealed a connection between the gut and liver, displaying that a long photoperiod in Taihe silkies leads to changes in gut microbiota composition and gene expression in the liver [13]. Despite growing evidence supporting microbiota–liver interactions, the mechanisms through which the gut–liver axis regulates energy metabolism in brooding TBSF remain incompletely elucidated.

In recent years, multi-omics approaches, including metabolomics, microbiomes, and transcriptomics, have been widely employed to decipher the regulatory role of the gut–liver axis in animal metabolism [14]. For example, Chen et al. employed multi-omics analysis to demonstrate that gut microbiota dysbiosis and disrupted hepatic lipid metabolism gene expression are key factors contributing to high-fat diet-induced fat deposition in chickens [15]. Similarly, Wu et al. applied integrated microbiome and transcriptome profiling to demonstrate that choline supplementation decreases gut microbial diversity and upregulates *APOA4* and *FABP1* gene expression in the liver, leading to liver steatosis and reduced egg production in laying hens [16]. However, the energy metabolism of brooding TBSF has not yet been investigated using a multi-omics framework.

To address research gaps in nutrient and energy metabolism in brooding layers, we utilized TBSF at the peak age of laying (224 days) for multi-omics analysis, including ultra-high-performance liquid chromatography–tandem mass spectrometry (UHPLC-MS/MS), 16S rRNA sequencing, and RNA sequencing (RNA-seq), to reveal the parameters underlying changes in nutrient and energy metabolism in the gut–liver axis. We anticipate that these findings will provide new insights into gut–liver axis-based mechanisms of energy metabolism and nutrient regulation in poultry production.

## 2. Materials and Methodology

### 2.1. Ethics Approval

This study was conducted in strict accordance with the references in the Guide for the Care and Use of Laboratory Animals. The procedure was reviewed and permitted by the Ethics Committee of Jiangxi Agricultural University (License Number: JXAULL-2024-05-1). To minimize suffering, all chickens were humanely euthanized at the end of the experiment.

### 2.2. Experimental Design and Animals

This study utilized 230 female TBSF chickens of uniform age (122 days) and body weight (0.9 kg), housed in an intensive scheme with ad libitum access to feed and water. Following an acclimatization period, the birds were separated at 193 days of age into two groups: a normal egg-laying group (NB), normally laying hens without any brooding behavior, set as the control group (*n* = 8), and a brooding group (BR30), which was broody for 30 days, serving as the experimental group (*n* = 8). All experimental TBSF chickens were raised in a free-range system under identical environmental conditions. The henhouse was fitted with drinkers, feeding troughs and nesting areas, together with an outdoor playground for free movement. Automatic video cameras were installed to continuously observe daily activities, egg laying and brooding behaviors of chickens. Residual feed for each group was weighed and recorded every morning after 24 h to serve as a weight baseline for subsequent calculations until the end of the experiment.

Total feed intake per group = initial feed weight − residual feed weight.

At that juncture, the average daily feed intake per hen was calculated by dividing the total feed consumed by the number of hens in each group.

### 2.3. Sample Collection

Eight healthy hens from the normal egg-laying group (NB) were randomly selected using random number generation for sampling. For the BR30 group, eight hens that exhibited continuous brooding behavior were sampled exactly on the 30th day of the brooding period. Before the laying hens were slaughtered, their body weight was measured, followed by the collection of blood directly from the wing vein. Blood samples were collected using tubes containing clot activator. The collected blood was allowed to coagulate at room temperature for 15 min before centrifugation. Samples were centrifuged at 3000 rpm (approximately 1500–1800× *g*) at 4 °C for 15 min. The separated serum was immediately stored at −80 °C for biochemical assays (TG and T-CHO) and untargeted metabolomics analysis (UHPLC-MS/MS). Subsequently, the hens were humanely euthanized via rapid cervical dislocation to induce instantaneous unconsciousness, followed by cervical exsanguination. Liver, duodenum, jejunum, ileum and cecal tissues were rapidly collected and weighed to calculate organ indices. At that moment, all tissues were immediately frozen in liquid nitrogen and stored in a −80 °C refrigerator. The liver tissue was stored for biochemical determination (TG and T-CHO), transcriptome sequencing, and quantitative Real-Time Polymerase Chain Reaction (qRT-PCR). The cecum contents were collected from the stored cecum for 16S cDNA analysis.

### 2.4. Feed Chemical Evaluation

The corn–soybean meal basal diet was formulated according to the 1994 guidelines provided by the National Research Council (NRC), as shown in Table 1, which lists the amount of ingredients and nutrients in the diet. By applying the 2020 China feed database, the metabolizable energy was calculated. The crude protein content was determined by the Kjeldahl method as outlined in the GB/T 6432-2018 of the China National Standard [17], though by using the Soxhlet extractor GB/T 6433-2006 in accordance with the China National Standard [18], the ether extract was assessed. Amino acids were identified by the GB/T 18246-2019 of the China National Standard [19]. Additionally, the ash content was determined by the GB/T 6438-2007 methodology specified in the China National Standard [20]. Total calcium and phosphorus were measured following the methods of B/T 6436-2018 and GB/T 6437-2018 [21,22].

### 2.5. Determination of Metabolic Tissue (Liver and Intestine)

After the laying hens were humanely euthanized by neck amputation, the liver and intestinal tissues were removed and separately placed on Petri dishes for weighing, and finally, the data were recorded for the calculation of the tissue indices (tissue index (%) = (tissue weight/body weight) × 100).

### 2.6. Determination of Serum and Liver Triglycerides and Total Cholesterol

The liver tissue sample is weighed (0.1 g), placed in phosphate-buffered saline, and then ground using high-speed, low-temperature homogenization treatment to prepare the homogenate. Subsequently, the total protein (BCA) of the liver tissue homogenate was measured according to the manufacturer’s instructions (Biyotime, Shanghai, China). Ultimately, concentrations of TG and T-CHO in the liver were measured according to the manufacturer’s protocol for the corresponding assay kits (Nanjing Jiancheng Bioengineering Research Institute, Nanjing, China). Moreover, the serum, stored at −80 °C, was allowed to thaw at room temperature, and the concentrations of TG and T-CHO were determined according to the reagent kit’s assays (Nanjing Jiancheng Bioengineering Research Institute, Nanjing, China).

### 2.7. 16S rRNA Sequencing and Analysis

Bacterial genomic DNA was extracted from cecal contents using the HiPure Stool DNA Kit (Guangzhou Meiji Biotechnology Co., Ltd., Guangzhou, China). DNA yield and purity were determined using a Nanodrop 2000 Spectrophotometer from Thermo Fisher Scientific (Waltham, MA, USA), with purity assessed by an A_260_/A_280_ ratio of 1.8–2.0 and an A_260_/A_230_ ratio ≥ 1.7. The V3–V4 hypervariable region of the bacterial 16S rRNA gene was amplified by PCR using the primer pair 341F (5′ CCTACGGGNGGCWGCAG 3′) and 806R (5′ GGACTACHVGGGTATCTAAT 3′). Each 25 μL reaction volume contained 12.5 μL of 2× Taq PCR Master Mix, 1 μL of each primer (10 μM), 2 μL of template DNA, and 8.5 μL of nuclease-free water. The PCR thermal program was: initial denaturation at 95 °C for 5 min; 27 cycles of 95 °C for 30 s, 55 °C for 30 s, and 72 °C for 45 s; followed by a final extension at 72 °C for 10 min.

Amplicons were purified using AMPure XP Beads (Beckman, Brea, CA, USA), quantified with a Qubit 3.0 Fluorometer, and validated on an ABI StepOnePlus Real-Time PCR System (Thermo Fisher Scientific (Waltham, MA, USA)). Equimolar purified amplicons were pooled and subjected to paired-end sequencing (PE250) on an Illumina NovaSeq 6000 platform (Illumina Inc., San Diego, CA, USA) at Gene Denovo Biotechnology Co., Ltd. (Guangzhou, China). A sequencing depth of at least 50,000 raw reads per sample was targeted, with an average of 62,300 high-quality reads obtained across all samples.

Raw data processing and quality control: raw reads were filtered to remove low-quality sequences (quality score < 20, length < 200 bp), reads with ambiguous bases, and chimeric sequences to obtain high-quality clean reads. Amplicon Sequence Variants (ASVs) were generated at 100% sequence similarity; alternatively, Operational Taxonomic Units (OTUs) were clustered at 97% similarity if used. Using the RDP, taxonomic annotation was completed against the Silva 138 database classifier with a confidence threshold of 0.7.

Subsequent analyses, including gut microbiota diversity, species composition, and LEfSe, were performed using Muscle (edition 3.8.31), FastTree (edition 2.1), QIIME (edition 1.9.1), and the R project combination, alpha value of 0.05 for the Kruskal–Wallis and Wilcoxon rank-sum tests, and an LDA score of 2.0 for highly significant features.

### 2.8. Untargeted Serum Metabolomics Analysis

Untargeted serum metabolomics analyses were performed using a Vanquish UHPLC system equipped with an Orbitrap Q ExactiveTM HF-X mass spectrometer, both from Thermo Fisher Scientific (Waltham, MA, USA), at Gene Denovo Biotechnology Co., Ltd. (Guangzhou, China). The raw data files generated by UHPLC-MS/MS were processed using Compound Discoverer 3.1, produced by Thermo Fisher Scientific (Waltham, MA, USA), to perform peak picking, peak alignment, and quantitation for individual metabolites. All peak intensities were normalized to the total positive and negative ion signal. Using the normalized data, molecular formulas were predicted based on precursor ions, adduct ions, and fragment ions. Peaks were annotated by matching against the mzCloud, mzVault, and MassList databases to obtain accurate qualitative and relative quantitative results. Principal Components Analysis (PCA), Orthogonal Projections to Latent Structures Discriminant Analysis (OPLS DA), differential metabolite identification, and Kyoto Encyclopedia of Genes and Genomes (KEGG) pathway enrichment analysis were performed using packages in the R environment.

### 2.9. RNA-Sequencing Analysis and Data Validation

Total RNA was extracted from chicken hepatic tissues using TRIzol reagent (Life Technologies, USA). Comprehensive RNA quality control was performed prior to library preparation: RNA integrity and genomic DNA contamination were assessed via agarose gel electrophoresis; RNA purity (A_260_/A_280_, A_260_/A_230_ ratio) was detected using a Nanodrop Micro Spectrophotometer (Thermo Fisher Scientific (Waltham, MA, USA)); RNA concentration was accurately quantified with a Qubit 2.0 Fluorometer; and RNA integrity number (RIN) was measured on an Agilent 2100 Bioanalyzer. Only RNA samples with RIN ≥ 6.8 were eligible for subsequent experiments.

Approximately 1 μg total RNA per sample was used as starting material for cDNA library construction. Ribosomal RNA (rRNA) was depleted to enrich messenger RNA (mRNA) owing to the high proportion of rRNA (>90%) in total RNA. mRNA was captured and enriched using mRNA Capture Beads, fragmented under high temperature, and then reverse-transcribed into first-strand cDNA. Second-strand cDNA synthesis was accompanied by end repair and A-tailing. After adapter ligation, target cDNA fragments were purified and screened using Hieff NGS^®^ DNA Selection Beads, followed by PCR amplification to construct sequencing libraries. All libraries were subjected to strict quality inspection, and qualified libraries were sequenced on the Illumina NovaSeq 6000 platform with a paired-end 150 bp (PE150) sequencing strategy. Sequencing work was completed by Gene Denovo Biotechnology Co., Ltd. (Guangzhou, China). After raw sequencing data were filtered to obtain high-quality clean reads, clean reads were aligned to the chicken reference genome assembly GRCg6a (*Gallus gallus*) using HISAT2 software (v2.2.1). Gene expression abundances were calculated via RSEM (v1.3.3) and StringTie (v1.3.1). Differentially expressed genes (DEGs) between the normal laying (NB) and 30-day brooding (BR30) groups were screened using DESeq2 (v1.20.0) with the criteria: |log_2_(fold change)| ≥ 1 and FDR < 0.05. Principal components analysis (PCA), Gene Ontology (GO) and Kyoto Encyclopedia of Genes and Genomes (KEGG) enrichment analyses of DEGs were implemented using R packages (v4.2.1).

Five representative DEGs (*APOC3*, *GPX3*, *PP1R3C*, *CYP51A1* and *ACAA1*) were randomly selected to validate the accuracy of RNA-seq results via quantitative Real-Time PCR (qRT-PCR). Total RNA was reverse-transcribed into cDNA using PrimeScript RT Master Mix (TaKaRa, Dalian, China) in a 10 μL reaction system: 2 μL 5×PrimeScript RT Master Mix, ≤500 ng total RNA, and RNase-free water supplemented to a final volume of 10 μL. The reaction program was 37 °C for 15 min, followed by 85 °C for 5 s to terminate reverse transcription. All specific primers were designed using NCBI Primer-BLAST (https://blast.ncbi.nlm.nih.gov/) and synthesized by Sangon Biotech (Shanghai, China).

qRT-PCR reactions were carried out on a StepOnePlus Real-Time PCR System (Thermo Fisher Scientific (Waltham, MA, USA)) with TB Green Premix Ex Taq II (TaKaRa). The 10 μL amplification system contained 5 μL 2×TB Green Premix Ex Taq II, 0.4 μL forward primer (10 μM), 0.4 μL reverse primer (10 μM), 0.2 μL 50×ROX Reference Dye, 1 μL cDNA template and 3 μL nuclease-free water. Cycling parameters: initial denaturation at 95 °C for 30 s; 40 cycles of 95 °C for 5 s and 60 °C for 30 s. Relative gene expression levels were calculated using the $2^{-∆∆C_{t}}$ method, with *β-actin* used as the housekeeping reference gene for normalization.

### 2.10. Statistical Data Analysis

Using IBM SPSS Statistics 26 (IBM Corp, Armonk, NY, USA), the experimental data were expressed as a mean ± standard error (mean ± SE) and subjected to independent sample testing. Data were analyzed using one-way analysis of variance (ANOVA). Differences with *p* < 0.05 were considered statistically significant, and *p* < 0.01 was considered highly significant. All graphs were generated using GraphPad Prism 8 software.

## 3. Results

### 3.1. Brooding Behavior Reduces Feed Intake and Body Weight

**Figure 1:**
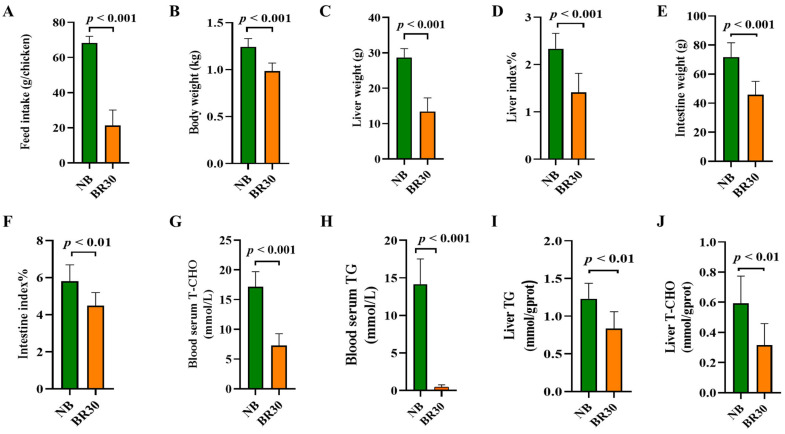
Effect of brooding in TBSF on (**A**) feed intake; (**B**) body weight; (**C**) liver weight; (**D**) liver index; (**E**) intestine weight; (**F**) intestine index; (**G**) blood serum (T-CHO); (**H**) blood serum (TG); (**I**) liver (TG); (**J**) liver (T-CHO). Figures show comparisons between the NB and B30 groups. Data are presented as means ± SEM (*n* = 8).

Feed intake and body weight changes were compared between the NB and BR30 groups. As shown in Figure 1A,B, the NB group had a significantly higher average daily feed intake (67.00 ± 0.85 g/bird/day, mean ± SE) than the BR30 group (21.79 ± 1.44 g/bird/day, mean ± SE; *p* < 0.001). Similarly, body weight gain was significantly greater in the NB group than in the BR30 group (*p* < 0.001). After 30 days of brooding, the average body weight of the BR30 group was 6.83 ± 1.64% lower than that of the NB group. Accordingly, the above results confirm that both feed intake and body weight were markedly reduced in the BR30 group relative to the NB group.

### 3.2. Brooding Induces Alterations of Key Metabolic Organs Indices and Systemic Lipid Profiles

After slaughtering, the abnormal cavity part was opened, and the liver and intestinal tissue were placed on Petri dishes for weighing and further analysis. The results showed a significant decline in liver weight and index in the BR30 group compared to the NB group (*p* < 0.001), as shown in Figure 1C,D. Consistently, the intestinal weight and index of the BR30 group were significantly decreased compared to the NB group (Figure 1E,F).

Additionally, the levels of total TG and T-CHO in the serum and liver during the brooding stages in TBSF are shown in Figure 1G–J. The results demonstrate that supplementation with BR30 significantly decreased serum T-CHO and TG levels compared to the NB group (*p* < 0.001). Likewise, the concentrations of liver T-CHO and TG were significantly lower in the BR30 group (*p* < 0.01) compared to the NB group. Consequently, the above results of the BR30 group compared with the NB group showed a significant atrophy of metabolic tissue and a decline of both liver and serum TG and T-CHO.

### 3.3. Brooding Alters the Gut Microbiome Structure

**Figure 2:**
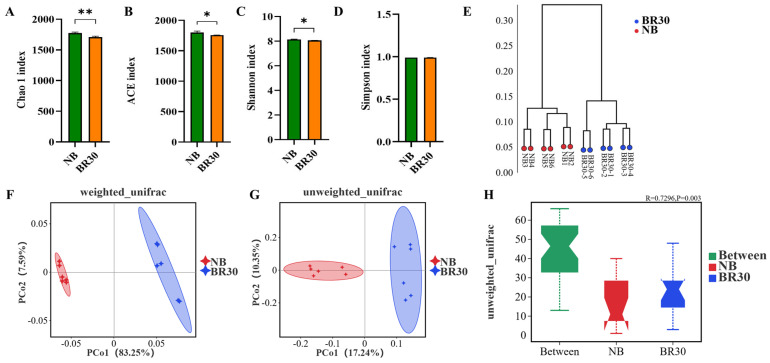
Effect of brooding on the diversity of gut microbiota in TBSF. Alpha diversity indices of gut microbial flora: (**A**) Chao1; (**B**) ACE; (**C**) Shannon; (**D**) Simpson. Beta diversity: (**E**) UPGMA cluster dendrogram according to the Bray distance matrix; (**F**) weighted unifrac-based and (**G**) unweighted unifrac-based principal coordinates analysis (PCoA) plots; (**H**) ANOSIM test based on the unweighted unifrac distance matrix. Figures show comparisons between the NB and B30 groups. * *p >* 0.05, ** *p* < 0.01.

The 16S rRNA sequencing was applied to determine the effect of brooding on the abundance and composition of cecal gut microbiota in laying hens of the BR30 group. The alpha diversity indices were applied to evaluate the evenness and richness of the microbial community. The three alpha diversity indices (Chao1, ACE, and Shannon) yielded significantly lower results in the BR30 group compared to the NB group, as shown in Figure 2A–D. At the Simpson level, no significant difference was detected. Next, beta diversity was employed to calculate the differences in bacterial community composition, and the overall analysis revealed a distinct structure of the gut bacterial flora between the NB group and the BR30 group (Figure 2E–H).

**Figure 3:**
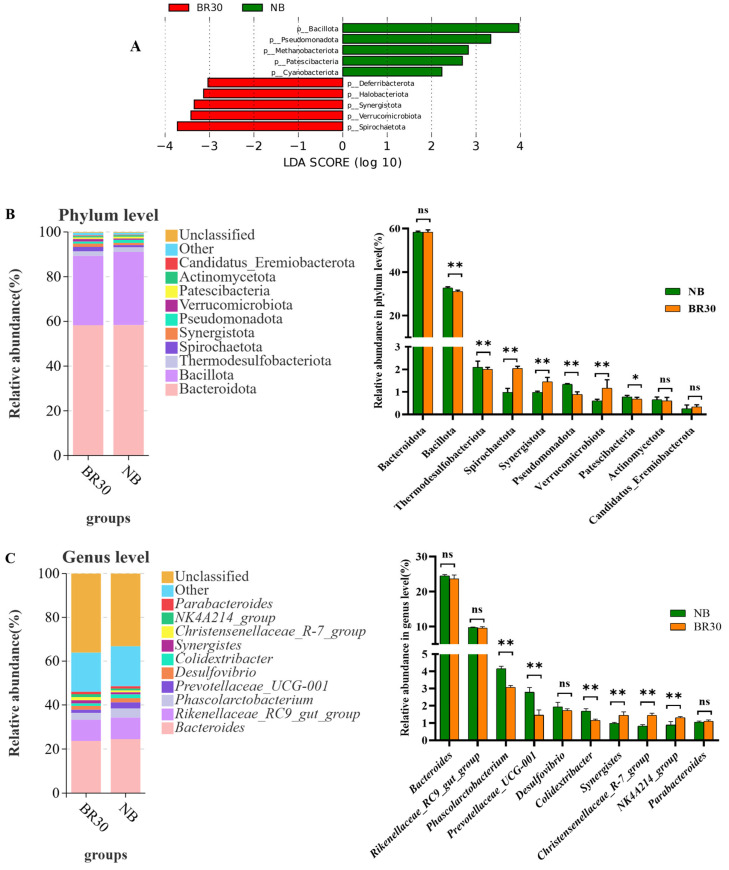
Differences in the taxonomic composition of cecum microbiota. (**A**) Linear Discriminant Analysis (LDA score > 4); (**B**) significant changes in relative abundance at the phylum level; (**C**) significant changes in relative abundance at the genus level. * *p* < 0.05, ** *p* < 0.01. Data are presented as means ± SEM (*n* = 6).

Formerly, Linear Discriminant Analysis Effect Size (LEfSe) was used to identify bacterial taxa characteristics and to compare differentially abundant taxa between groups. The analysis revealed a significantly clearer separation of taxa between the groups. As shown in the results, the phyla *Bacillota*, *Pseudomonadota*, *Methanobacteriota*, *Patescibacteria*, and *Cyanobacteriota* were significantly enriched in the NB group compared to the BR30 group; the abundance of phyla *Spirochaetota*, *Synergistota*, *Verrucomicrobiota*, *Deferribacterota*, and *Halobacteriota* was enriched in the BR30 group, suggesting a clear separation of phyla between them in the gut microbial structure of TBSF (Figure 3A). In addition, the alteration in the gut microbiota composition at the phylum and genus levels was also determined by taxonomic analysis. At the phylum level, *Bacteroidetes* plus *Bacilliota* were the highest phyla in both groups (Figure 3B). However, several taxa, including *Spirochaetota*, *Synergistota*, and *Verrucomicrobiota*, showed significant increases in relative abundance in the BR30 group compared to the NB group (*p* < 0.05 or *p* < 0.01). In contrast, *Bacillota*, *Thermodesulfobacteriota*, *Pseudomonadota*, and *Patescibacteria* were significantly higher in the NB group. Other taxa, such as *Actinobacteria* and *Caulobacterales*, also showed differences but were less definite. At the genus level, *Bacteroides* and *Rikenellaceae* RC9 gut group were the most abundant genera in both groups, with no statistically significant difference (ns) observed between the groups. Likewise, in the NB group, the abundance of *Phascolarctobacterium*, *Prevotellaceae_UCG-001*, and *Colidextribacter* was significantly higher compared to the BR30 group, while the abundance of the genus *Synergistetes*, *Christensenellaceae R-7 group*, and *NK4A214_group* was considerably elevated in the BR30 group compared to the NB group, as seen in Figure 3C.

### 3.4. Brooding Perturbs the Serum Metabolome

**Figure 4:**
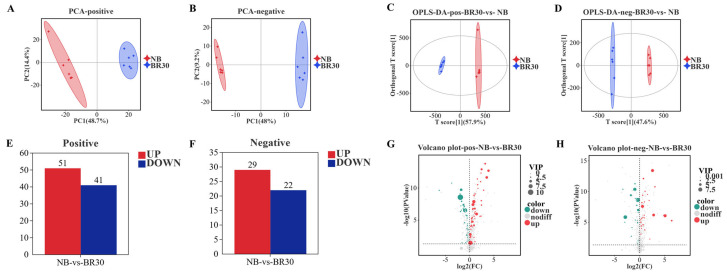
Changes in serum metabolic profiles in positive and negative ions. (**A**,**B**) Principal components analysis (PCA); (**C**,**D**) Orthogonal Partial Least Squares–Discriminant Analysis (OPLS-DA); (**E**,**F**) differential metabolites bar plot; (**G**,**H**) differential metabolites volcano plot (*n* = 6). Figures show comparisons between the NB and B30 groups.

**Figure 5:**
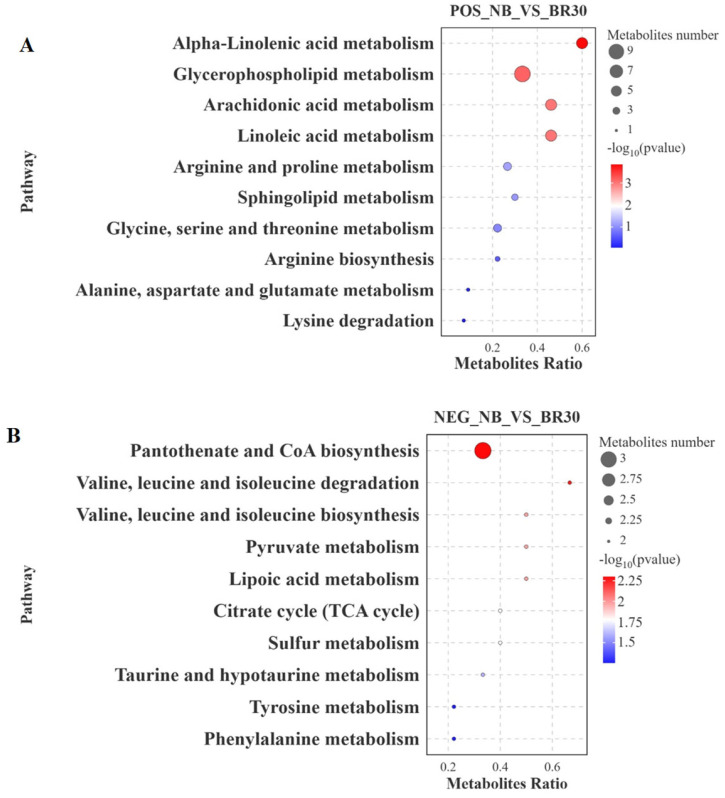
KEGG enrichment analysis of differential metabolites: (**A**) positive ion mode; (**B**) negative ion mode.

Untargeted metabolomics analysis was useful to classify the differential serum metabolites between the NB and BR30. As shown in Figure 4A–D, the tight clustering of samples in PCA and OPLS-DA analyses within each group in both negative and positive ion modes demonstrates high intra-group homogeneity, supporting their suitability for subsequent analyses. Based on the screening criteria of VIP > 1 and *p* < 0.05, a total of 143 differential metabolites were identified. Among these metabolites, 92 metabolites were detected in the positive ion mode, 51 were upregulated, and 41 were downregulated (Figure 4E). The remaining 51 metabolites were detected in the negative ion mode, with 29 being upregulated and 22 being downregulated (Figure 4F). As well, the volcano plot analysis in both positive and negative (Figure 4G–H) revealed that 143 metabolites were differentially accumulated (|log_2_(FC)| ≥ 1, adjusted *p*- value ≤ 0.05) between the groups. The top 20 serum metabolites with the highest variable importance in projection (VIP) values in positive and negative ion modes are presented in Supplementary Figure S1A,B. The abbreviations of the screened differential metabolites are listed in Supplementary Tables S1 and S2. In positive ion mode, comparison between the NB group and the BR30 group revealed four differential metabolites: trimethylamine N-oxide, stigmastane-3,6-dione, Pro-Leu, and propionylcarnitine (Car (3:0)). These metabolites were downregulated in the NB group but upregulated in the BR30 group. In contrast, in negative ion mode, four differential metabolites, phenylsulfate, N-acetyl-2-carboxybenzenesulfonamide, monobutyl phthalate, and nitrofurazone, showed the opposite trend: they were upregulated in the BR30 group and downregulated in the NB group. Furthermore, KEGG pathway enrichment analysis demonstrated that the differential serum metabolites in positive ion mode were notably enriched in six pathways, including alpha-linoleic acid metabolism, glycerophospholipid metabolism, arachidonic acid metabolism, and linoleic acid metabolism. In the negative ion mode, nine enriched pathways were notably included: pantothenate and CoA biosynthesis; valine, leucine, and isoleucine degradation; valine, leucine, and isoleucine biosynthesis; pyruvate and lipoic acid metabolism; glucagon signaling pathway; citric acid cycle (TCA cycle); and sulfur metabolism (Figure 5A,B). Therefore, the above results express changes in differentially metabolized compounds between the groups.

### 3.5. Brooding Reprograms the Hepatic Transcriptome

**Figure 6:**
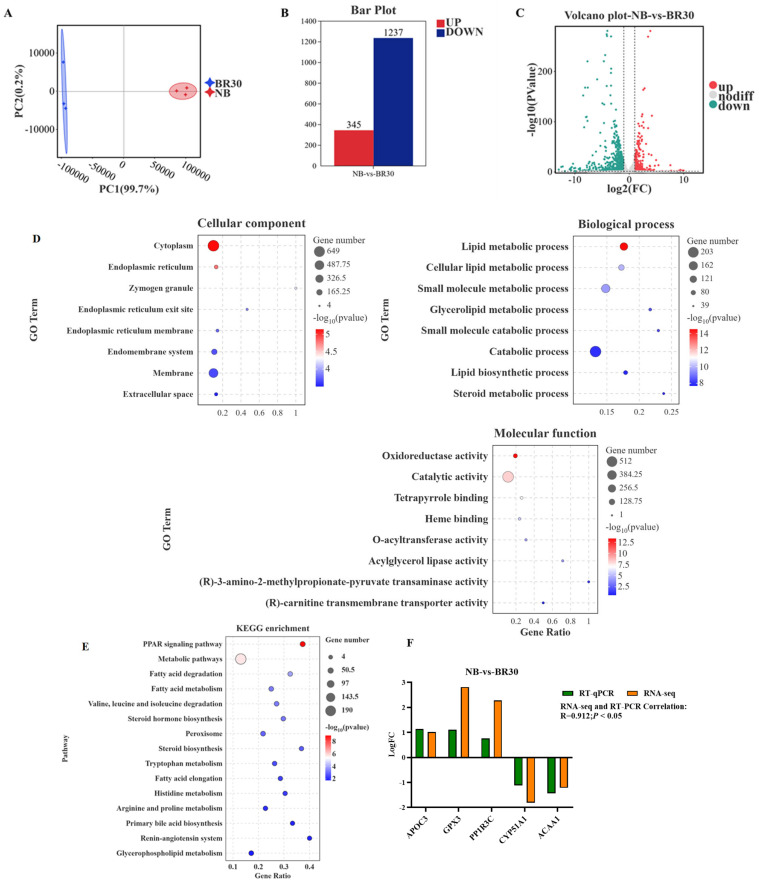
Changes in gene expression in the liver of the TBSF: (**A**) principal components analysis (PCA); (**B**,**C**) differential metabolites bar plots and volcano plot, respectively; (**D**) GO enrichment Cellular Component, Biological Process, Molecular Function, respectively; (**E**) KEGG enrichment analysis pathway; (**F**) gene validation (qRT-PCR (*n* = 3)). Figures show comparisons between the NB and B30 groups.

Differentially Expressed Genes (DEGs) were identified by RNA-seq analysis and screened with a threshold of false discovery rate *p* < 0.05 and |log_2_(FC)| ≥ 1. The PCA plot revealed obvious separation between the NB and BR30 groups, indicating that brooding treatment induced substantial alterations in gene expression. A total of 1582 DEGs were identified from 17,482 screened genes. Among them, 345 genes were significantly upregulated, and 1237 genes were considerably downregulated. Specifically, genes, including *COX2*, *ND2*, *APOA1*, *ND1*, *COX1*, *AGXT2*, *LPL*, *ATP8*, and *PRODH*, were significantly upregulated in the BR30 group compared to the NB group, whereas *PIP4P1*, *PPT2*, *INPPL1*, *PCK1*, *GALM*, and *ACO1* was significantly downregulated (*p* < 0.05), as shown in Figure S2. These differences reflect transcriptional changes occurring during brooding in TBSF. The validity of RNA-seq results was verified by randomly selecting five DEGs (*ACAA1*, *APOC3*, *CYP51A1*, *PPP1R3C*, and *GPX3*), and expression levels were determined using the qRT-PCR method (Figure 6F).

Moreover, to further understand the possible functions involved in DEGs, we performed GO and KEGG analysis on DEGs between the NB and BR30 groups. The results of GO analysis were described in the Biological Process (BP), Cellular Components (CC), and Molecular Function (MF) categories based on the Q-value. The top 10 most significantly enriched GO terms are presented in a bubble plot in Figure 6D. The lipid and amino acid-related metabolism processes, like lipid metabolism, cellular lipid metabolism, small molecules metabolism, glycerolipid metabolism, small molecules catabolic process, catabolic process, lipid biosynthesis, steroid metabolism, organic hydroxy compound metabolic process, carboxylic acid metabolism, alcohol metabolism, oxoacid metabolism, and fatty acid catabolic process, were significantly enriched in the BR30 group.

In addition, to examine the molecular response of TBSF layers during the brooding phase, KEGG pathway enrichment analysis was performed. As shown in Figure 6E, the bubble plot displays the top 15 enriched pathways ranked by *p*-value. The dot plots represent the number of DEGs and significance levels, where larger and red-colored dots indicate pathways with more DEGs and lower *p*-value DEGs are significantly enriched in pathways closely related to the lipid metabolism and the amino acid metabolism, respectively, such as the PPAR signaling pathway, metabolic pathway, fatty acid degradation, fatty acid metabolism, valine, leucine, and isoleucine degradation, steroid hormone biosynthesis, peroxisome, steroid biosynthesis, Tryptophan metabolism, fatty acid elongation, histidine metabolism, and arginine and proline metabolism.

### 3.6. The Correlation Analysis of Microbiome and Metabolome

**Figure 7:**
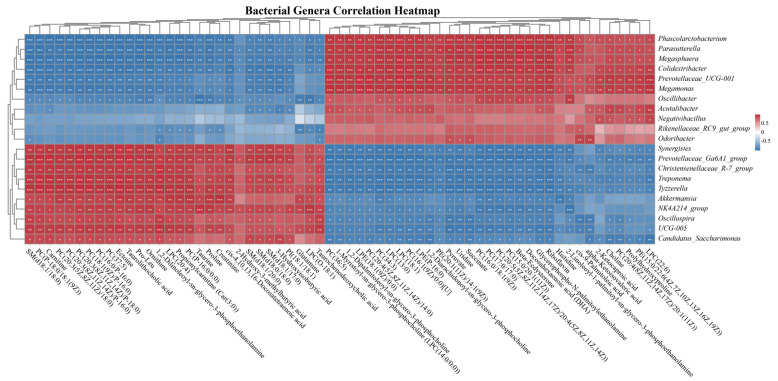
Correlation heatmap of differential metabolites and differential gut microbial genera, Pearson’s coefficient: red squares (positive) and blue squares (negative) correlations. Correlation strength was categorized based on the absolute value of the correlation coefficient (|*r*|): * indicates a weak correlation (|*r*| < 0.50), ** indicates a moderate correlation (0.50 ≤ |*r*| < 0.75), and *** indicates a strong correlation (0.75 ≤ |*r*| ≤ 1.00).

To determine the significantly correlated genera and their associated metabolic pathway, we utilized Pearson’s correlation analysis to reveal significant relationships between 63 differential serum metabolites and 21 gut flora. Detailed results are shown in Figure 7. Specifically, lipid classes, including various Phosphatidylcholines (PC, PC-, and LPC), glycerophospholipids, and Sphingomyelin (SM(d18:1/17:0), showed strong positive (red)/negative (blue) correlations with 14 to 17 bacterial genera, such as *Phascolarctobacterium*, *Treponema*, *Parasutterella*, *Megasphaera*, *Megamonas*, *Synergistes*, *NK4A214_group*, and *Christensenellaceae_R-7_group*. Similarly, organic acids and derivatives, such as pyruvate, succinate, and branched-chain amino acid (BCAA) metabolites, were strongly correlated with 16 genera.

### 3.7. The Correlation Analysis of Metabolome and Transcriptome

**Figure 8:**
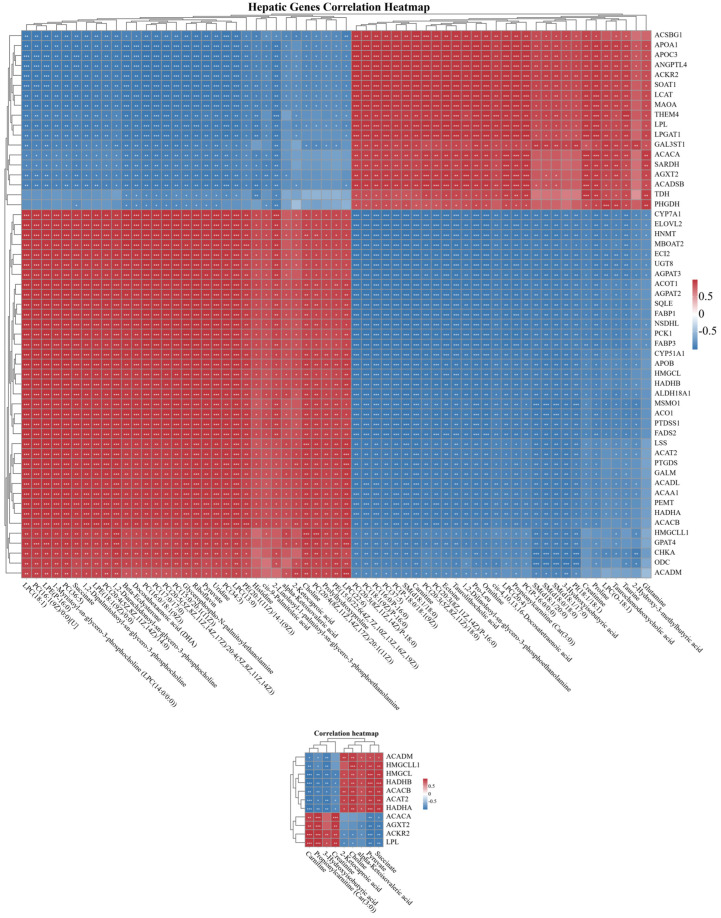
Correlation heatmap of differential metabolites and genes. Pearson’s coefficient: red squares (positive) and blue squares (negative) correlations. Correlation strength was categorized based on the absolute value of the correlation coefficient (|*r*|): * indicates a weak correlation (|*r*| < 0.50), ** indicates a moderate correlation (0.50 ≤ |*r*| < 0.75), and *** indicates a strong correlation (0.75 ≤ |*r*| ≤ 1.00).

To investigate the relationship between serum metabolite levels and differentially expressed genes in the liver during brooding of TBSF, Pearson’s correlation analysis was performed on the differentially expressed metabolites and genes. A total of 63 differentially expressed metabolites were selected from the serum metabolome, and 55 differentially expressed genes enriched in representative bioprocesses related to energy metabolism (lipid metabolism and organic acid metabolism) from the liver transcriptome were selected from the GO analysis results. Correlation analysis was then performed between the two omics datasets. The results shown in Figure 8 indicate a significant correlation between differentially expressed metabolites and genes. Thirteen genes involved in lipid synthesis (e.g., *ACSBG2*, *PEMT*, *ACOT1*, *NSDHL*, *SQLE*, *ACAT2*, *ELOVL2*, *FADS2*, *CYP51A1*, *FABP1*, *AGPAT2*, *APOB*, and *LPL*) were extremely positively correlated with the same lipid metabolites. This integrated analysis underscores a significant gut–liver axis, where shifts in the gut microbiome are associated with concurrent changes in host systemic metabolism and hepatic gene expression, particularly in lipid and amino acid pathways.

## 4. Discussion

Broodiness is a natural behavior in poultry, characterized by hens sitting on eggs to incubate them, which is governed by complex neuroendocrine mechanisms [23]. This instinct significantly reduces feed intake and alters energy metabolism, leading to economic losses in the commercial poultry industry. TBSF is one of the breeds known for its strong natural brood instincts [24]. During the brooding period, the hen noticeably reduces her feed intake and spends more time incubating the clutch of eggs, leaving the nest for about 20–30 min to eat, which interferes with their energy metabolism [24,25]. Consistent with this, Jehl et al. found that low dietary intake in chickens affected energy expenditure and altered lipid and protein metabolism to generate ATP [26]. Ultimately, reduced feed intake in birds impairs bird health and subsequent production performance. Similarly, a study in broilers found that restricting feed intake significantly decreases the average daily weight gain, directly impacting productivity and weakening health [27].

In the present study, significant differences in energy metabolism were observed between the BR30 group and NB groups of TBSF laying hens. Notably, non-broodiness is the characteristic phenotype of NB hens: these hens maintain stable egg production throughout the trial without displaying broody behavior. Broodiness induced by elevated prolactin stops oviposition, whereas non-broody hens sustain continuous laying performance [24]. In accordance with our findings, the marked loss of feed intake, body weight, and atrophy of metabolic tissues (intestinal and liver) observed in the BR30 group significantly indicates an overall decline in energy reserve and reveals a shift in carbohydrate metabolism to lipid utilization. For example, studies by Richards et al. and Meyer et al. have shown that starvation-induced weight loss disrupts energy homeostasis by shifting metabolism from carbohydrates toward lipids and amino acids, thereby enabling animals to mobilize energy for survival [28,29].

The liver is a vital organ that regulates many homeostatic functions. As the predominant hepatic parenchymal cells, hepatocytes regulate hepatic biochemical activities and metabolic processes, including TG and T-CHO metabolism [30]. During energy-demanding states such as starvation, brooding, molting, or fasting, the liver shifts its metabolism to favor lipid utilization over carbohydrate metabolism, which is reflected by changes in liver TG and T-CHO levels [24,31]. Similarly, our findings show that during the brooding stage of TBSF (BR30), both serum and hepatic TG and T-CHO levels significantly decreased, indicating a major shift in lipid metabolism driven by a switch from carbohydrate to lipid use to meet energy needs. These results align with previous research that highlights how TG and T-CHO levels are regulated according to the diet, such as a study in domestic pigeons showing that excess dietary fat intake leads to increased TG and T-CHO levels in the liver and causes obesity in pigeons [32].

In addition, the gut–liver axis is a bidirectional communication axis that modulates energy and nutrient metabolism in animals. This process begins in the intestine, the critical site for nutrient digestion and absorption. Specifically, the small intestine is responsible for the efficient breakdown of dietary feed.

Concurrently, the cecum, a key site for microbial fermentation, can contribute to this metabolic load by enhancing the extraction of calories and SCFAs from the diet, further promoting energy storage and weight gain. Thus, in animals, the liver, intestine, and cecum are crucial organs in governing nutrient absorption and energy utilization.

The microbiome was studied to explore the impact of brooding for 30 days in broody Silky fowl. Accordingly, numerous studies demonstrated that the richness, evenness, and composition of the cecum microbiota are closely associated with animal metabolism. For instance, *Bacteroidota* and *Bacillota* are the two major bacterial phyla of the animal gut microbiota; fluctuations in these bacteria may lead to changes in energy metabolism [6]. The findings of this study on 16S rRNA sequencing revealed distinct compositional differences in the cecal microbiota between the two groups. The alpha diversity of cecal microbiota differed significantly between the NB and BR30 groups (*p* < 0.05) for Chao1, ACE and Shannon indices, whereas no significant differences were found in the Simpson index. We assessed beta diversity via three approaches: UPGMA cluster dendrogram based on the Bray–Curtis distance matrix, unweighted and weighted UniFrac-based principal coordinates analysis (PCoA), and ANOSIM test relying on the unweighted UniFrac distance matrix. Collectively, these analyses revealed obvious clustering of cecal microbial communities, indicating functional divergence of the cecal microbiome between the two groups.

Moreover, under conditions of nutrient limitation, the gut microbiota exhibits a marked adaptive shift at both the phylum and genus levels, reflecting a strategic reorientation toward fermentative metabolism. At the phylum level, the relative abundances of *Spirochaetota*, *Synergistota*, and *Verrucomicrobiota* were significantly higher in the BR30 group than in the NB group. These phyla are functionally adapted to utilize alternative energy sources: *Spirochaetota* and *Synergistota* are capable of fermenting amino acids, while *Verrucomicrobiota* primarily degrade host mucins and complex dietary fibers. A previous study by Harwood demonstrated that the availability of branched-chain amino acids, including L-isoleucine, L-valine, and L-leucine, significantly supports the survival of *Spirochaeta* under nutrient-limited conditions [33]. Under starvation, these organisms ferment such amino acids to generate adenosine 5′ triphosphate (ATP) and branched-chain fatty acids, thereby sustaining energy supply [33,34]. Additionally, studies in mice have shown that high-fat and high-sugar diets significantly reduce the abundance of *Verrucomicrobiota*, including the key mucin-degrading genus *Akkermansia* [35].

This phylum-level shift is coherently reflected at the genus level, where taxa associated with fiber and protein fermentation, *Synergistetes*, *NK4A214_group*, and the *Christensenellaceae R-7 group* were enriched. The study by Cesare showed that *Synergistetes* was significantly higher in chickens fed a low-diet than in those fed a high-diet, fermenting amino acids and proteins, gaining an advantage when dietary carbohydrates decline, and protein metabolism becomes more important for energy release [36]. In addition, a study by Tavella et al. in Italian elderly demonstrated that the *Christensenellaceae* R 7 group is a beneficial genus associated with improved metabolic health and profiles. In the present study, conducted in TBSF chicken, this genus showed distinct abundance patterns between the BR30 and NB group [37]. The increasing abundance of this genus improves gut health and reduces colonization by certain pathogens, such as *Campylobacter* and *Salmonella*, in the organisms [38]. Still, research by Luigi et al. [39,40] in humans showed that the abundance of the *Christensenellaceae* family is consistently associated with weight loss and low BMI.

Concurrently, the increase in abundance of *Phascolarctobacterium*, a genus in the NB group, was positively related to body fat mass and weight gain. Previous studies have shown that an increase in the abundance of *Phascolarctobacterium* definitely correlated with body fat mass, weight gain, plasma leptin, increased triglyceride concentrations, and increased glucose tolerance after receiving a high-fat diet [41,42]. Collectively, the previous study is similar to our finding that the BR30 group shows a highly significant level of *Synergistetes*, *Christensenellaceae R-7 group*, and *NK4A214_group* genera and a decline of *Phascolarctobacterium* genera. So, the shift in genera emphasizes a microbial strategy to enhance fermentative metabolism, compensating for reduced dietary energy intake by generating host-usable metabolites (e.g., SCFAs) from indigestible fibers. This remodeling of the microbiota supports the gut–liver axis by shifting the pool of microbial metabolites available for hepatic processing, as noted in Heiss et al.’s review on gut microbiota-dependent modulation of energy metabolism [11].

Metabolomics is a method utilized for the discovery and identification of metabolites within cells and tissues, playing a dynamic role in biological research. As established in previous research, insufficient feed intake or fasting can trigger a fundamental shift in energy metabolism, moving away from glucose utilization and towards the catabolism of lipids and amino acids, and induce extensive changes in the abundances of lipids and amino acids in the blood [43]. For instance, kinetic studies in neonatal piglets by Robert et al. revealed that the acute (0–2 h) and chronic (2–12 h) phases of fasting significantly change glucose metabolism and increase the levels of amino acid metabolism, such as isoleucine, leucine, phenylalanine, and valine in plasma concentration [44]. The fasting-induced shift towards lipid metabolism is equally pronounced. Servane et al., using the king penguin model, demonstrated that glucose regulates lipid metabolism during fasting, leading to a significant increase in lipolysis and fatty acid oxidation, concurrent with a decrease in glucose metabolism [45]. Jo et al. also discovered that in mice fed a high-fat diet, the blood serum lysine and threonine levels were considerably decreased [46]. These metabolic principles are highly relevant to the unique physiological state of brooding hens [46], adding to previous studies that clarify that a brooding hen has a higher resting metabolic rate due to heat production for egg incubation, which leads to a strong effect on energy expended [47,48]. The review by Hu et al. [24] also shows that blood glucose levels in brooding silky layers are higher than in non-brooding silky layers. Collectively, these previous findings provide a coherent mechanistic backdrop for our own research, which similarly demonstrates that brooding in layers increases the levels of lipids, amino acids, and energy metabolism.

In this study, transcriptome analysis was performed to identify candidate genes and pathways strongly associated with brooding in layers, particularly in energy metabolism regulation. We utilized liver tissue as a vital organ for regulating different metabolic processes. As previous research has shown, under starvation conditions, the liver changes its carbohydrate metabolism to lipid and amino acid in order for the animal to survive. This process is regulated by a complex molecular network that manages hepatic carbohydrate to lipid and amino acid metabolism. In our study, RNA-seq analysis identified 1582 DEGs in the liver of hens, with 1237 downregulated and 345 upregulated genes—reflecting a transcriptional program focused on energy conservation and catabolism. Upregulated DEGs included key genes involved in oxidative phosphorylation (*ND1*, *ND2*, *COX1*, *ATP8*) and amino acid catabolism (*PRODH*), while downregulated genes included those linked to lipid synthesis (*ACADM*, *INPPL1*) and glucose metabolism (*PIP4P1*). The increase in the oxidative phosphorylation gene response to higher ATP requirements enhances mitochondrial biogenesis, often driven by increased energy demand or reliance on anaerobic respiration [49]. Moreover, *PRODH* genes break down proline (an amino acid) from body protein, like muscle, into glutamate to serve as an alternative energy source when glucose and fatty acids are depleted during animal starvation. A study by Bideyan et al. revealed *PRODH* gene is upregulated during the fasting period of the animals, and the liver catabolizes amino acids, which contributes to gluconeogenesis [50]. The *ACADM* gene critical role in making enzymes that break down medium-chain fatty acids for energy. During periods of lower feed intake, energy metabolism prioritizes stored glycogen before utilizing long-chain fatty acids, a more efficient pathway. The concurrent downregulation of *ACADM* reduces the oxidation of medium-chain fatty acids, thereby conserving energy and helping hens maintain systemic energy balance [51]. Collectively, the evidence from previous research and our current study demonstrates that brooding triggers transcriptional changes in genes involved in key metabolic pathways: oxidative phosphorylation, amino acid oxidation, and lipid oxidation.

GO enrichment analysis indicated that it exhibited significant enrichment with lipid metabolic processes (e.g., glycerolipid metabolism, fatty acid catabolism) and amino acid catabolic processes—consistent with the depletion of hepatic lipids and the need to use amino acids as energy substrates. KEGG pathway enrichment further highlighted the PPAR signaling pathway (a major controller of lipid catabolism), fatty acid degradation, and BCAA degradation as the top-enriched pathways. The PPAR signaling pathway, in particular, plays a critical role in triggering genes involved in fatty acid β-oxidation, clarifying the reduced hepatic TG levels in BR30 hens [52]. For example, downregulation of ACADM genes, involved in the fatty acid β-oxidation pathway, further suppresses lipid storage, confirming that available lipids are directed toward energy production rather than accumulation. This transcriptional profile confirms the liver’s role as the driver of metabolic adaptation, orchestrating the shift from anabolism (lipid storage for production) to catabolism (lipid/amino acid breakdown for incubation) [51].

Results of Pearson’s correlation analysis showed prominent correlative relationships among intestinal microorganisms, serum metabolites and hepatic genes, further confirming that the gut–liver axis modulates metabolic alterations caused by brooding. Lipid metabolites (e.g., phosphatidylcholines, sphingolipids) were positively correlated with 23–25 bacterial genera (including *Synergistes* and *Christensenellaceae R-7 group*), indicating that microbial metabolites directly influence systemic lipid homeostasis. Organic acids (e.g., pyruvate, succinate) and BCAA metabolites were also correlated with 19–25 genera, linking microbial fermentative metabolism to host energy production. The study in humans showed *Phascolarctobacterium* was positively correlated with α-linolenic acid and negatively correlated with phosphatidylcholine metabolites [53,54].

At the gut–liver crossing point, microbial metabolites (e.g., SCFAs, TMAO, Phenylsulfate) are transported via the hepatic portal vein to the liver, where they regulate the expression of genes involved in lipid and amino acid metabolism. For example, SCFAs (produced by Spirochaeta and Synergistota in BR30) can activate hepatic PPAR signaling, enhancing fatty acid oxidation [55]. Conversely, hepatic bile acids—secreted into the gut to aid lipid absorption—may further modulate microbiota composition, creating a bidirectional regulatory loop. The strong correlations between serum metabolites (e.g., Propionylcarnitine) and hepatic genes (e.g., *ACSBG2*, *FABP1*) further confirm that the gut–liver axis synchronizes metabolic processes to support brooding: the gut microbiota adapts to produce metabolites that signal the liver to shift from anabolism to catabolism, ensuring energy is available for incubation. For example, Wu et. al.’s study revealed that FABP1 is activated by the PPAR signaling pathway, which is similar to our finding [56].

This study has some limitations. We used pairwise correlation analysis to explore the interrelationships among hepatic genes, serum metabolites and gut bacteria. Correlation analysis cannot confirm causal interactions among these variables. In addition, microbial network analysis and microbiome functional prediction were not performed in this manuscript. Such in-depth analyses will be conducted in our future studies.

## 5. Conclusions

In conclusion, this multi-omics study demonstrates that brooding behavior in TBSF chickens triggers systemic physiological adaptations, including weight loss, tissue atrophy, altered gut microbiota, changes in hepatic gene expression and serum metabolites, with coordinated changes observed across these biological layers. Furthermore, our findings reveal that energy metabolism shifts from carbohydrate utilization to lipids and amino acid catabolism; key taxa (e.g., *Synergistetes*, *Christensenellaceae R-7 group*) and pathways (e.g., PPAR signaling, β-oxidation) sustain homeostasis under limited food intake. Overall, brooding prioritizes survival maintenance over somatic growth. Future work will examine short-term and long-term brooding stages to map progressive remodeling and its impacts on health and productivity.

## Figures and Tables

**Table 1:** Composition and nutrient levels of the basal diet (%, air-dry basis).

Nutrients in the Diet	Content
Ingredients (g/kg)	
Corn	64.5
Soybean meal	23.5
Fish meal	1.5
CaHPO_4_	1.5
Limestone	8.0
NaCl	0.3
L-lysine monohydrochloride (L-lysine ≥ 78.8%)	0.1
Feed grade DL-methionine (DL-methionine ≥ 98.5%)	0.2
Choline chloride (≥50%)	0.1
Premix ^1^	0.3
Total	100.0
Nutrient levels ^2^	
Metabolizable energy, MJ/kg	11.56
Crude protein	16.35
Ether extract	3.08
Ash	3.16
Calcium	3.49
Total phosphorus	0.58
Available phosphorus	0.39
Lysine	0.91
Methionine	0.51
Threonine	0.57

^1^ The premix provided the following per kg of the diet: VA 12,000 IU, VD_3_ 4400 IU, VE 85 mg, VK_3_ 5 mg, VB_1_ 4 mg, VB_2_ 15 mg, VB_3_ 65 mg, VB_5_ 21 mg, VB_6_ 7 mg, VB_12_ 0.035 mg, folic acid 3 mg, biotin 0.35 mg, Fe (as ferrous sulfate) 75 mg, Mn (as manganese sulfate) 25 mg, Cu (as copper sulfate) 2.5 mg, Zn (as zinc sulfate) 115 mg, I (as potassium iodide) 1.0 mg, Co (as sodium selenite) 0.48 mg, Se (as sodium selenite) 0.35 mg, Cr (as sodium selenite) 0.2 mg. ^2^ Metabolizable energy and available phosphorus amino were measured standards, while the others were calculated standards.

## Data Availability

All raw data supporting the findings of this study are provided in the main article or the Supplementary Materials. Additional datasets are available from the corresponding author upon reasonable request.

## Supplementary Materials

The following supporting information can be downloaded at: https://www.mdpi.com/article/10.3390/ani16152416/s1, Figure S1. Enrichment analysis of metabolites (*n* = 6); (A) positive (B) negative heatmap of metabolites. Table S1. Screening results of differential metabolites between the NB group and the BR30 group under positive ion mode. Table S2. Screening results of differential metabolites between the NB group and the BR30 group under negative ion mode. Figure S2. Clustering analysis of differentially expressed genes. Table S3. Phenotypic changes in Taihe Black-Bone Silky Fowls during the 30-day brooding period. Table S4. List of genes and their primer sequences. Table S5. KEGG pathway enrichment of differential metabolites. Table S6. Differential mean abundance of bacterial genera in Taihe silky fowl across brooding stages.

## Abbreviations

ATP, adenosine triphosphate; TG, total glycerides; T-CHO, total cholesterol; KEGG, Kyoto Encyclopedia of Genes and Genomes; GO, Gene Ontology; PCA, principal components analysis; FC, fold change; VIP, variable importance in projection; UH-PLC-MS/MS, ultra-high performance liquid chromatography mass spectrometer/mass spectrometer.
