# Supplementary material for: Integrated Multi-Omics Analysis Reveals Molecular Features Associated with Energy Metabolism Adaptations in Brooding Taihe Black-Bone Silky Fowls
Source: Animals (Basel). 2026 Aug 5;16(15):2416. doi: 10.3390/ani16152416 (PMC13465929; doi:10.3390/ani16152416)
Supplement: Supplementary file 1 [file animals-16-02416-s001.zip › animals-4360743-supplementary.pdf]

Figure S1

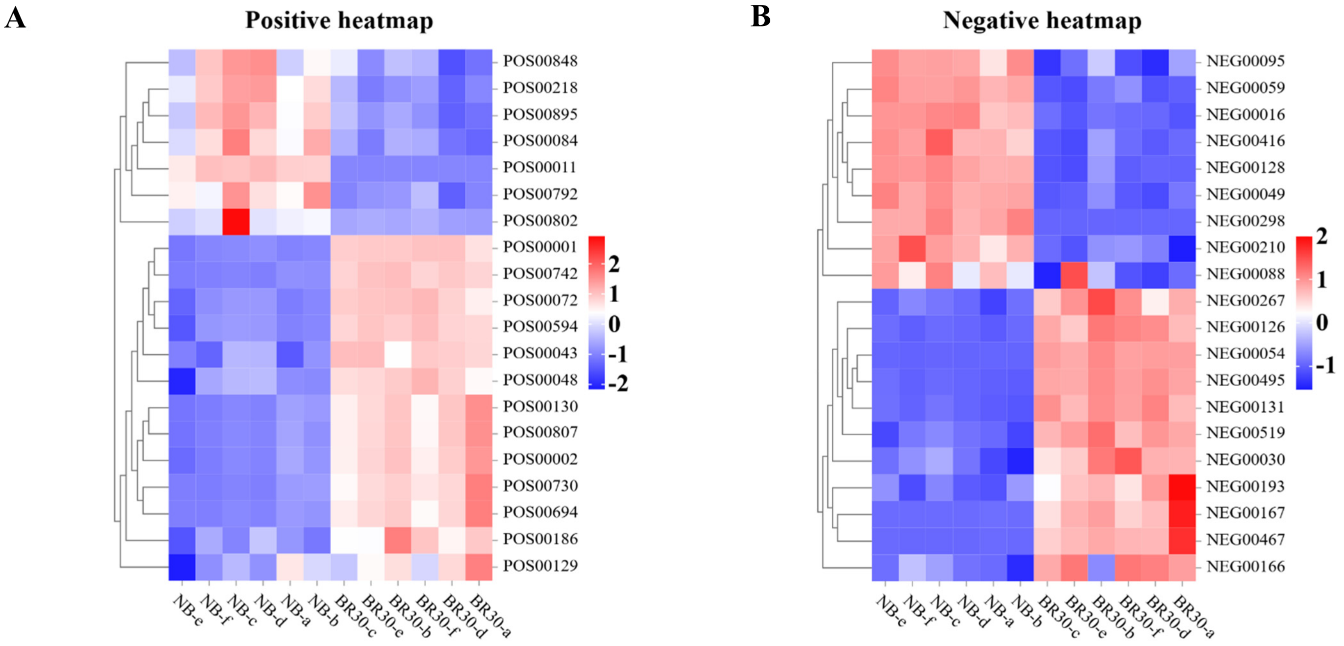

Figure S1. Enrichment analysis of metabolites (n = 6); (A) positive (B) negative heatmap of metabolites

Table S1. Screening results of differential metabolites between the NB group and the BR30 group under positive ion mode.

Number	ID	MS2 name	VIP	*P-value*
1	POS00001	Trimethylamine N-oxide	1.0530	3.26E-11
2	POS00002	SM(d18:1/17:0)	1.7736	8.00E-07
3	POS00011	Riboflavin	1.6957	2.28E-10
4	POS00043	Taurochenodeoxycholic acid	1.1058	5.93E-06
5	POS00048	Proline	2.7631	8.91E-05
6	POS00072	Propionylcarnitine (Car(3:0))	1.4676	2.43E-08
7	POS00084	Prolylhydroxyproline	2.0793	0.000239
8	POS00129	Palmitoyl sphingomyelin	7.6166	0.034536
9	POS00130	SM(d18:1/18:0)	5.2675	1.11E-06
10	POS00186	Sph(t18:0)	1.1280	0.000120
11	POS00218	Sulfatide(d18:1/17:0)	1.0746	3.86E-05

12	POS00594	Pro-Leu	1.3762	3.06E-08
13	POS00694	SM(d18:1/20:0)	1.9245	2.36E-06
14	POS00730	SM(d16:1/24:1(15Z))	1.3319	2.67E-06
15	POS00742	Stigmastane-3,6-dione	1.9615	1.03E-11
16	POS00792	PE(20:1(11Z)/14:1(9Z))	1.5757	7.56E-05
17	POS00802	PE(18:2(9Z,12Z)/20:4(8Z,11Z,14Z,17Z))	1.5321	0.034194
18	POS00807	SM(d18:0/18:0)	1.7819	1.07E-06
19	POS00848	Tsugaric_acid_B	2.0121	0.008525
20	POS00895	PE(15:0/22:6(4Z,7Z,10Z,13Z,16Z,19Z))	1.3895	0.000257

Table S2. Screening results of differential metabolites between the NB group and the BR30 group under negative ion mode.

Number	ID	MS2 name	VIP	*P-value*
1	NEG00016	Pantothenic acid	1.0602	1.33E-09
2	NEG00030	Taurine	2.3180	9.88E-06
3	NEG00049	Pyruvate	9.4927	2.32E-09
4	NEG00054	Phenysulfate	5.6102	3.73E-14
5	NEG00059	Uridine	2.2870	2.42E-09
6	NEG00088	alpha-Ketoisovaleric acid	1.2562	0.043259
7	NEG00095	cis-9-Palmitoleic acid	2.5966	1.16E-05
8	NEG00126	Taurolithocholic acid	1.3741	2.79E-09
9	NEG00128	Succinate	1.0867	3.37E-09
10	NEG00131	Monobutyl phthalate	1.0610	2.86E-10
11	NEG00166	Tauro-beta-muricholic acid	1.2877	0.000843
12	NEG00167	PI(18:1/18:1)	3.0590	5.60E-06
13	NEG00193	Prostaglandin I2 (PGI2)	1.1027	0.000105
14	NEG00210	Lithospermoside	1.2071	5.06E-06
15	NEG00267	cis-4,10,13,16-Docosatetraenoic acid	1.7040	1.67E-06
16	NEG00298	N-Acetyl-D-galactosamine 4-sulfate	1.4984	4.19E-11
17	NEG00416	Methyl 4-formyl-3-hydroxy-2-naphthoate	1.1431	2.73E-07
18	NEG00467	PI(16:0/18:1)	1.0284	7.30E-07
19	NEG00495	N-Acetyl-2-carboxybenzenesulfonamide	2.8991	7.70E-13

20	NEG00519	Nitrofurazone	5.6253	2.62E-08

Figure S2 Genes clustering heatmap

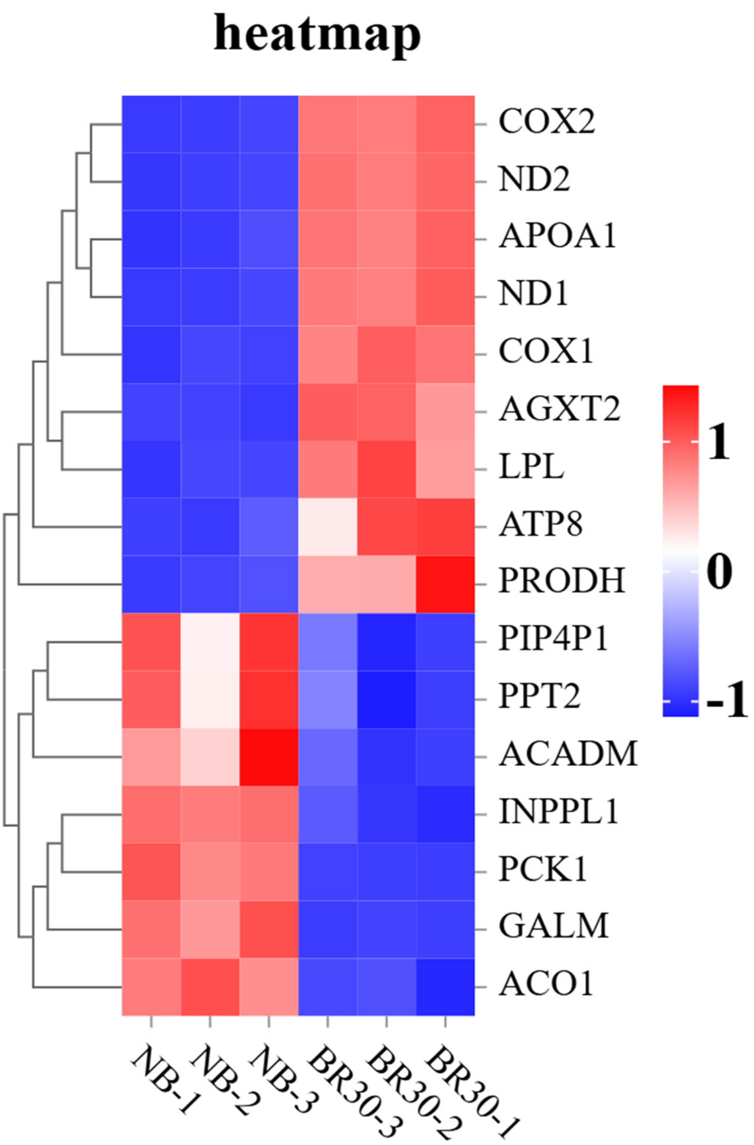

Figure S2. Clustering analysis of differentially expressed genes

Table S3. Phenotypic changes in Taihe Black-Bone Silky Fowls during the 30-day brooding period

**Item**	**NB**	**BR30**	***P*<value**
Feed intake ((g/chicken)	68.29±0.65	21.35±1.47	<0.001
Weight change (kg)	1.24±0.03	0.98±0.03	<0.001

Liver weight (g)	28.72±0.88	14.25±1.71	<0.001
Liver index %	2.33±0.12	1.41±0.16	<0.001
Intestine weight (g)	71.82±3.48	45.80±3.79	<0.001
Intestine index%	5.81±0.31	4.49±0.29	<0.01
Liver TG (mmol/gprot)	1.23±0.07	0.92±0.13	<0.01
Liver T-CHO (mmol/gprot)	0.49±0.53	0.34±0.12	<0.01
Blood serum TG (mmol/L)	14.03±1.11	0.46±0.10	<0.001
Blood serum T-CHO (mmol/L)	17.16±0.90	0.73±0.70	<0.001

Table S4. List of genes and their primer sequences

**Genes symbols**	**Forward primer (5'_3')**	**Reverse primer (3'_5')**
APOC3	TCCATCCTGCTCCTGCTCATC	CACCTTCTTCACCACCACTTCTG
ACAA1	TGGCATGGCCTGTGGAATAA	TGTCTTTTGGTTGCCCTCGT
CYP51A1	GCCAGACAGCATAGGGGAA	ACCTCAGCCGATTTTCACTAA
PPP1R3C	AGCCCCGCAGAAGGAAAATGAT	AGTGGTGGTGAATGGGCTAAG
GPX3	TCCAGAAAGGGGATGTGAACG	GGAGGGCAGGAGTTCTTCAGG

Table S5. KEGG pathway enrichment of differential metabolites

**Pathway**	**Pathway ID**	***P*<value**	**Q-value**
Pantothenate and CoA biosynthesis	ko00770	0.00490	0.12220
Valine, leucine, and isoleucine degradation	ko00280	0.00530	0.12220
Valine, leucine, and isoleucine biosynthesis	ko00290	0.01033	0.12220
Pyruvate metabolism	ko00620	0.01033	0.12220
Arachidonic acid metabolism	ko00590	0.00086	0.00790
Arginine and proline metabolism	ko00330	0.06032	0.41472
Lipoic acid metabolism	ko00785	0.01033	0.12220
Glucagon signaling pathway	ko04922	0.01033	0.12220
Citrate cycle (TCA cycle)	ko00020	0.01678	0.13238
Sulfur metabolism	ko00920	0.01678	0.13238
cAMP signaling pathway	ko04024	0.01678	0.13238
Taurine and hypotaurine	ko00430	0.02454	0.15839
AMPK signaling pathway	ko04152	0.04422	0.17441

Metabolic pathways	ko01100	0.08515	0.18637
Glycolysis / Gluconeogenesis	ko00010	0.08662	0.18637
alpha-Linolenic acid metabolism	ko00592	0.00013	0.00244
Retrograde endocannabinoid signaling	ko04723	0.00013	0.00244
Glycerophospholipid metabolism	ko00564	0.00063	0.00790
Linoleic acid metabolism	ko00591	0.00086	0.00790
Sphingolipid metabolism	ko00600	0.07641	0.42252
Vitamin digestion and absorption	ko04977	0.08802	0.42254
ABC transporters	ko02010	0.09548	0.42250
Riboflavin metabolism	ko00740	0.28501	0.50567

Table S6. Differential mean abundance of bacterial genera in Taihe silky fowl across brooding stages.

**Genus**	**NB-mean**	**BR30-mean**	**Domain**	**Phylum**
Bacteroides	24.47571	23.6647	Bacteria	Bacteroidota
Rikenellaceae_RC9_gut_group	9.739321	9.589888	Bacteria	Bacteroidota
Phascolarctobacterium	4.153542	3.072654	Bacteria	Bacillota
Prevotellaceae_UCG-001	2.787175	1.458859	Bacteria	Bacteroidota
Desulfovibrio	1.927242	1.721204	Bacteria	Thermodesulfobacteriota
Colidextribacter	1.685257	1.157381	Bacteria	Bacillota
Synergistes	0.983351	1.451107	Bacteria	Synergistota
Christensenellaceae_R-7_group	0.82015	1.444771	Bacteria	Bacillota
NK4A214_group	0.882541	1.308381	Bacteria	Bacillota
Parabacteroides	1.051294	1.106238	Bacteria	Bacteroidota
Prevotellaceae_Ga6A1_group	0.519442	1.497021	Bacteria	Bacteroidota
Faecalibacterium	0.948029	1.011456	Bacteria	Bacillota
Mediterraneibacter	1.018291	0.864859	Bacteria	Bacillota
Megamonas	1.482935	0.241697	Bacteria	Bacillota
Treponema	0.467753	1.173534	Bacteria	Spirochaetota
Oscillibacter	0.897118	0.576798	Bacteria	Bacillota
Alistipes	0.647478	0.821147	Bacteria	Bacteroidota
Akkermansia	0.300637	0.997976	Bacteria	Verrucomicrobiota

Acutalibacter	0.618442	0.523977	Bacteria	Bacillota
Parasutterella	0.681725	0.345763	Bacteria	Pseudomonadota
